# An integrated approach of bioinformatic prediction and in vitro analysis identified that miR-34a targets *MET* and *AXL* in triple-negative breast cancer

**DOI:** 10.1186/s11658-018-0116-y

**Published:** 2018-10-24

**Authors:** Shadan Hajalirezay Yazdi, Mahdi Paryan, Samira Mohammadi-Yeganeh

**Affiliations:** 1 Department of Cellular and Molecular Biology, Faculty of Advanced Sciences & Technology, Pharmaceutical Sciences Branch, Islamic Azad University, Tehran, Iran; 2 Department of Research and Development, Production and Research Complex, Pasteur Institute of Iran, Tehran, Iran; 3 Cellular and Molecular Biology Research Center, Shahid Beheshti University of Medical Sciences, Tehran, Iran; 4 Department of Biotechnology, School of Medicine, Shahid Beheshti University of Medical Sciences, Tehran, Iran; 5 Department of Biotechnology, School of Advanced Technologies in Medicine, Shahid Beheshti University of Medical Sciences, Tehran, Iran

**Keywords:** Breast cancer, miRNA-34a, *AXL*, *MET*, Real-time PCR

## Abstract

**Background:**

Breast cancer is the most prevalent cancer among women, and *AXL* and *MET* are the key genes in the PI3K/AKT/mTOR pathway as critical elements in proliferation and invasion of cancer cells. MicroRNAs (miRNAs) are small non-coding RNAs regulating the expression of genes.

**Methods:**

Bioinformatic approaches were used to find a miRNA that simultaneously targets both *AXL* and *MET* 3′-UTRs. The expression of target miRNA was evaluated in triple-negative (MDA-MB-231) and HER2-overexpressing (SK-BR-3) breast cancer cell lines as well as normal breast cells, MCF-10A, using quantitative real-time PCR. Then, the miRNA was overexpressed in normal and cancer cell lines using a lentiviral vector system. Afterwards, effects of overexpressed miRNA on the expression of *AXL* and *MET* genes were evaluated using quantitative real-time PCR.

**Results:**

By applying bioinformatic software and programs, miRNAs that target the 3′-UTR of both *AXL* and *MET* mRNAs were determined, and according to the scores, miR-34a was selected for further analyses. The expression level of miR-34a in MDA-MB-231 and SK-BR-3 was lower than that of MCF-10A. Furthermore, *AXL* and *MET* expression in SK-BR-3 and MDA-MB-231 was lower and higher, respectively, than that of MCF-10A. After miR-34a overexpression, *MET* and *AXL* were downregulated in MDA-MB-231. In addition, *MET* was downregulated in SK-BR-3, while *AXL* was upregulated in this cell line.

**Conclusions:**

These findings may indicate that miR-34a is an oncogenic miRNA, downregulated in the distinct breast cancer subtypes. It also targets *MET* and *AXL* 3′-UTRs in triple-negative breast cancer. Therefore, it can be considered as a therapeutic target in this type of breast cancer.

## Background

Breast cancer is one of the most common diseases among women and has a high incidence rate worldwide [1]. Carcinogens, life style, nutrition, and demographic factors are among the causes of breast cancer. These can change hormonal status and gene expression, both of which affect signaling pathways. Growth factor receptors have a marked effect on the proliferation and cell survival of breast cells and other epithelial tissues [2, 3]. Receptor tyrosine kinases (RTKs) that control the development of mammalian cells are among such receptors. Studies show that dysregulation of RTKs is related to breast cancer initiation. Therefore, they can be used as targets for breast cancer treatment [4]. The PI3K/AKT pathway, which receives signals from cell surface RTKs, has a pivotal role in various cancers, and its dysregulation can trigger cancerous transformation. The phosphatidylinositol-3-kinase pathway is activated upon binding of growth factors to RTKs. Activating mutations in the pathway’s genes can initiate cancer-like phenotypes such as increased proliferation, survival, and migration [5]. AXL is a cell surface RTK whose ligand is Gas2. Upon binding, AXL activates the PI3K/AKT signaling pathway. Therefore, it has a great impact on proliferation, cell survival, and metastasis [6–8]. AXL is highly resistant to chemotherapy and therefore it is a suitable target for drug design. It is related to angiogenesis and metastasis [9, 10], and its mutations result in tumor formation and expansion [11]. MET signaling is related to pro-apoptotic and anti-apoptotic cascades. Treatments using anti-MET agents are effective in basal-like breast cancer and triple-negative breast cancer (TNBC). Clinical research has indicated the role of *MET* in breast cancer [12]. In fact, *AXL* and *MET* are known oncogenes, and different studies show that they have roles in progression of breast cancer.

Many studies have been conducted to block dysregulated signaling pathways by inactivating oncogenes to avoid the use and side effects of chemotherapy. MicroRNAs (miRNAs) are small non-coding RNAs that regulate expression of genes, i.e. they act as tumor suppressor miRNAs or oncogenic miRNAs. After transcription, miRNAs control gene expression. They inhibit or suppress translation of mRNAs by binding to their 3′-UTR [13, 14]. Although miRNAs are still new molecules in the biological world, scores of studies have been conducted to elucidate the relationship between miRNAs and various cancers such as breast cancer. These studies also aimed to show that miRNAs are suitable sources for diagnosis and management of breast cancer. Therefore, they can be used as biomarkers for cancer diagnosis/prognosis as well as targets for cancer therapies.

Advances in bioinformatic algorithms and programs have led to the development of software applications with capability of predicting miRNAs targeting different mRNAs. To reduce the rate of errors in bioinformatic methods, we used several software applications simultaneously to generate more reliable results. In addition, we examined the accuracy of our predictions using quantitative real-time PCR (RT-qPCR).

Therefore, in this study, we first applied bioinformatics tools to predict miRNAs targeting *AXL* and *MET* 3′-UTR simultaneously. Then, we investigated the expression of the candidate miRNA and the two oncogenes (*AXL*, *MET*) in MDA-MB-231 and SK-BR-3 cell lines. The results were compared with microarray data. Finally, we analyzed the effects of overexpression of the target miRNA on the expression of the target genes to determine whether the miRNA can be used for targeted therapy of breast cancer. Eliminating cancer cells will probably be more effective by finding a miRNA that simultaneously targets two oncogenes.

## Methods

### Bioinformatic prediction of miRNAs

Different software applications and bioinformatic databases such as miRWalk [15], TargetScan [16], miRanda [17], DIANA-microT-CDS [18], and PicTar [19] were used to predict miRNAs targeting *AXL* and *MET* 3′-UTRs. First, the sequences of the genes were retrieved from GenBank, NCBI. Then, the targeting miRNAs were predicted using the miRNA databases, and those with the highest scores were selected. Afterwards, among the high score miRNAs, those targeting both genes were selected for further analyses.

### Cell lines and cell culture

MDA-MB-231 (triple-negative invasive ductal breast cancer), SK-BR-3 (Her-2 overexpressing breast cancer cell line), and MCF-10A (normal breast cells) were purchased from the National Cell Bank of Iran (Pasteur Institute of Iran, Tehran). MDA-MB-231 and SK-BR-3 cells were cultured in Dulbecco’s modified Eagle medium (DMEM) supplemented with 10% fetal bovine serum (FBS). MCF-10A cells were cultured in DMEM supplemented with 10% horse serum (HS) and other supplements necessary for its culture. All cells were incubated at 37 °C in a humidified atmosphere and 5% CO_2_. All cell culture media and supplements were purchased from Gibco, USA.

### Primer design

Primer design for *AXL* (NM_021913.4), *MET* (NM_001127500.2), and *ACTB* (beta-actin as a housekeeping gene, NM_001101.4) was performed using AlleleID6 and Oligo7. miR-34a and SNORD47 (as housekeeping small nuclear RNA) primers were designed based on a previously published article by Mahammadi-Yeganeh et al. [20].

### Total RNA extraction, cDNA synthesis, and quantitative real-time PCR

Total RNA was extracted from cell lines using RNX-Plus (CinnaClone, Iran). The quality and quantity of the extracted RNA were determined by agarose gel electrophoresis and spectrophotometry, respectively. cDNA synthesis was performed using random hexamers and RevertAid Reverse Transcription Enzyme (Fermentas, Leon-Rot, Germany). miRNA cDNA synthesis was performed using RT-Stem loop primers. RT-qPCR was used to determine the expression of *MET* and *AXL*. RT-qPCR reactions were performed in a final volume of 20 μl containing 0.4 μl of 50X passive dye ROX, 0.8 μl of forward and reverse primers (10 μM), 2 μl of cDNA, and 10 μl of 2X RealQ Plus Master Mix Green (Amplicon, Denmark). The RT-qPCR thermal profile was as follows: 95 °C for 10 min for enzyme activation, followed by 40 cycles of 94 °C for 10 s, and 60 °C for 40 s. At the end of the amplification cycles, melting temperature analysis was performed by a slow increase in temperature (0.3 °C/s) from 60 to 95 °C. The relative fold change in the expression of the target genes was determined using the 2^-ΔΔCT^ method. All tests were performed in triplicate using a StepOne instrument (Applied Biosystems, USA).

### Viral packaging in HEK 293 T cells and virus collection

HEK 293 T cells were used as host cells for lentiviral production using the CaPo_4_ protocol [21]. Briefly, cell transfection was performed using plasmids including pMD2G (encoding the VSV-G envelope protein), psPAX (packaging vector), and either PLEX-JRED-TurboGFP-miR-34a (PJTG-miR-34a) or PLEX-JRED-TurboGFP (PJTG) control vector. Sixteen hours after transfection, the medium was replaced with fresh medium. Afterwards, supernatant containing viruses was harvested for three consecutive days (every 24 h) and stored at 4 °C until use.

### Concentration of viruses and cell transduction

After centrifugation at 2500 g and filtration with 0.45 nm filters to remove cell debris, 50% PEG 8000 (final concentration of 5%) and 5 M NaOH (final concentration of 0.15 M) were mixed with viruses and incubated at 4 °C in a shaker rolling incubator overnight. The next day, tubes containing viruses were centrifuged at 4100 g for 20 min at 4 °C. The supernatant was removed and the precipitate was dissolved in cold DMEM media. MDA-MB-231, SK-BR-3, and MCF-10A cell lines were cultured in 24-well plates and transduced by concentrated viruses using polybrene (4 μg/μl). Since polybrene is fatal for cells, the medium was replaced with fresh medium after 6–8 h. For selection of transduced cells, after 24 h, DMEM+ 10% FBS was replaced with complete medium containing 1 μg/ml puromycin. Fluorescent microscopy was used to measure transduction efficiency.

### RNA extraction from transduced cells and RT-qPCR

After 72 h of puromycin treatment, almost 90% of the cells were fluorescent. Then, RNA extraction and cDNA synthesis were performed as previously mentioned in section of Methods. RT-qPCR was performed to evaluate the effects of miR-34a overexpression on the target genes according to the protocol mentioned before in Methods.

### Statistical analysis

RT-qPCR results (before and after miR-34a overexpression) were analyzed with StepOne software and REST 2009. GraphPad Prism6 was used to perform statistical analyses and draw graphs.

## Results

### miRNA prediction for *AXL* and *MET*

**Fig. 1:**
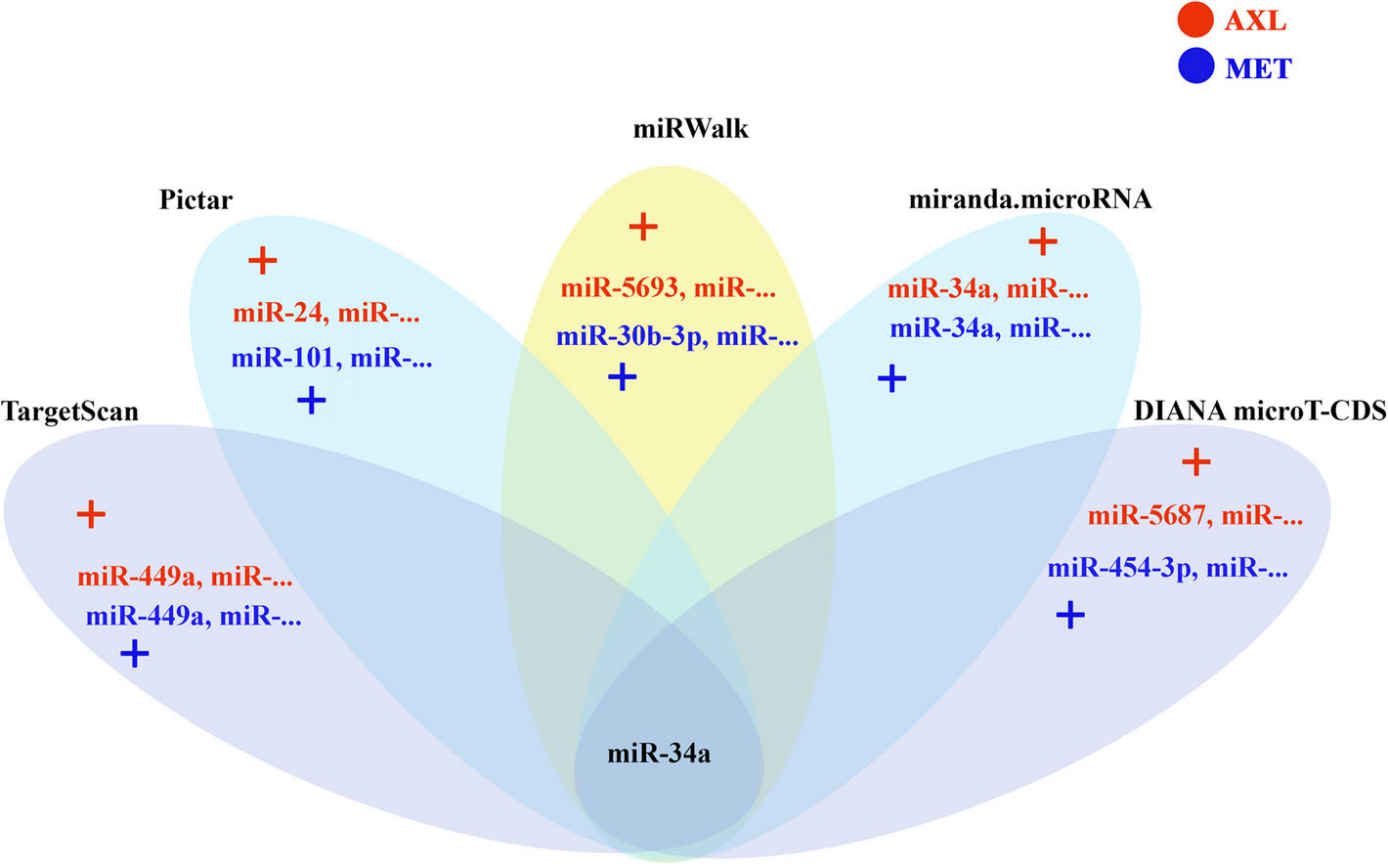
Predicted miRNAs targeting both AXL (red) and MET (blue)

Software applications and databases such as TargetScan, miRanda, PicTar, miRWalk, and DIANA-microT-CDS were used to predict miRNAs targeting *AXL* and *MET-*3′-UTRs. Among the predicted miRNAs, miR-34a had the highest score and targeted both *AXL*- and *MET* 3′-UTRs. Therefore, it was selected for further analyses (Fig. 1).

### *MET*, *AXL*, and miR-34a expression analysis in the cell lines

**Fig. 2:**
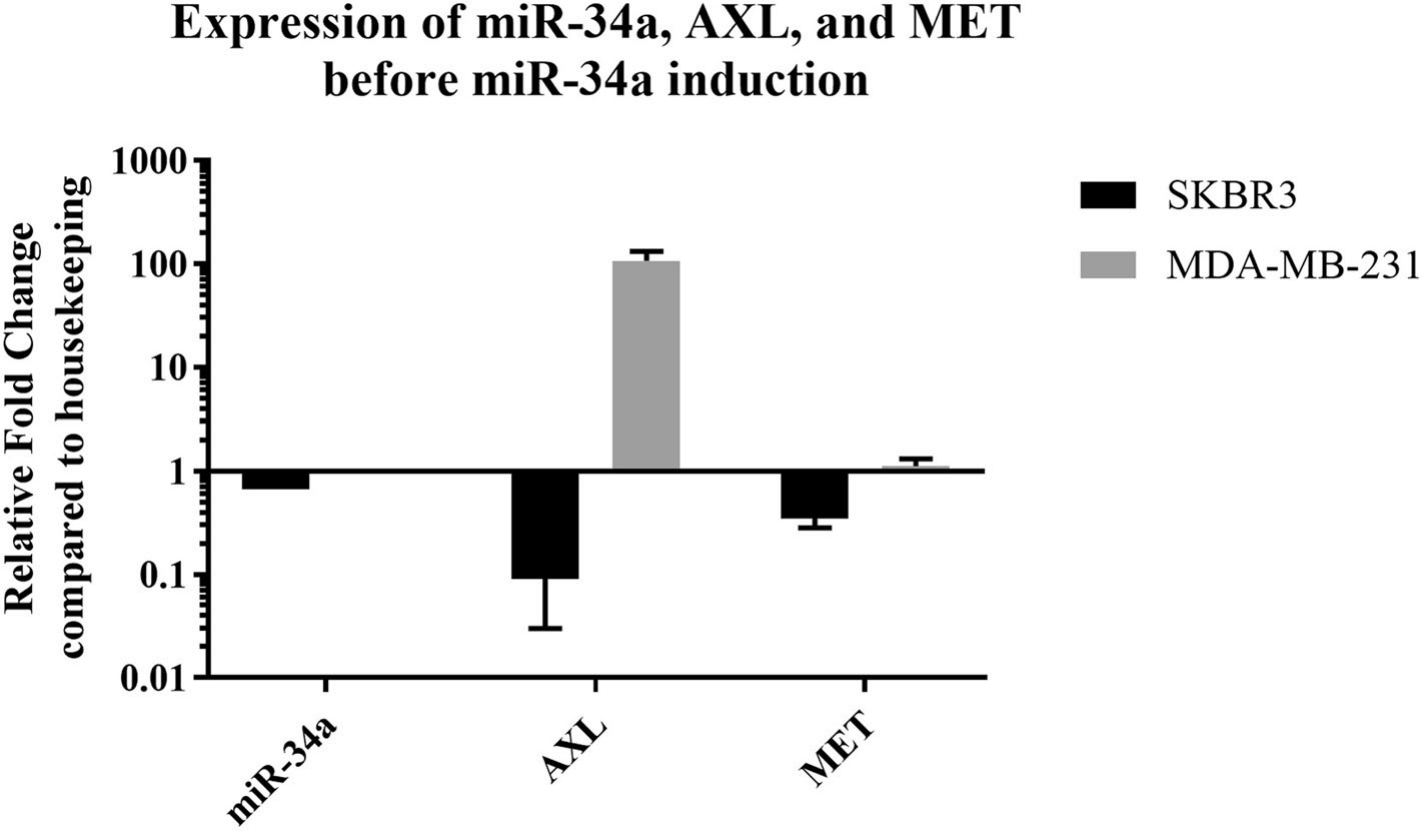
Expression of miR-34a, *AXL*, and *MET* in MDA-MB-231 and SK-BR-3 cell lines before miR-34a induction

Using the RT-qPCR method, the expression of the genes and predicted miRNA was assessed in MDA-MB-231 and SK-BR-3 compared to the MCF-10A cell line as a normal control (Fig. 2).

### HEK 293 T transfection, virus production, and cell line transfection

**Fig. 3:**
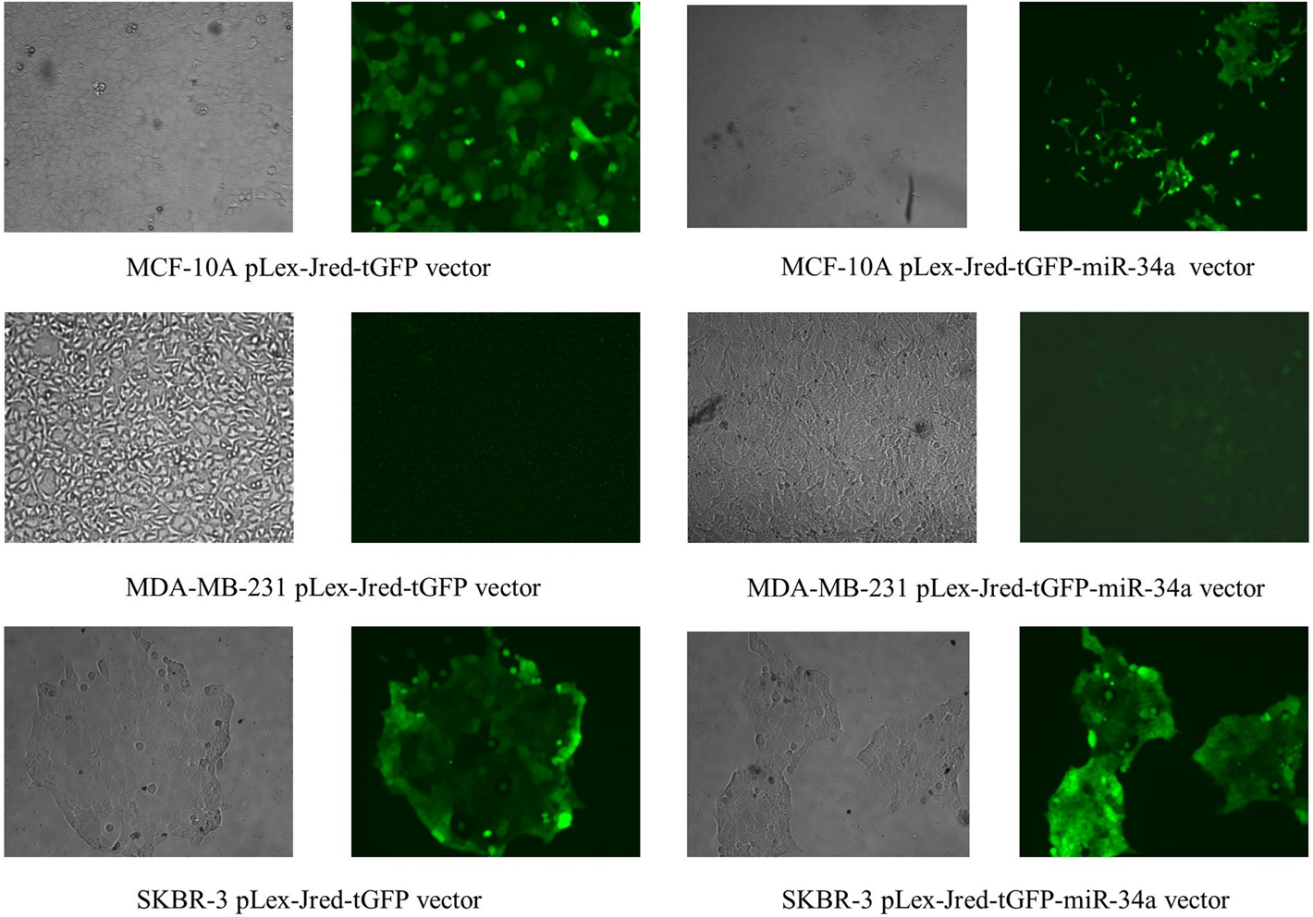
Light and fluorescent microscopy picture of MCF-10A, MDA-MB-231, and SK-BR-3 cells transduced with viruses containing control vector or miR-34a

Using the plasmids psPAX2, pMD2.G, PJTG (control plasmid), and PJTG-miR-34a, HEK 293 T cells were transfected to produce miR-34a or a control vector containing the virus. After transfection, cell culture supernatant, containing packaged viruses, was collected for 3 days. The PEG-concentrated control and miR-34a-containing viruses were then used to transduct the cell lines. Figure 3 shows the cell lines after transduction.

### *AXL* and *MET* expression after miR-34a induction in cell lines

**Fig. 4:**
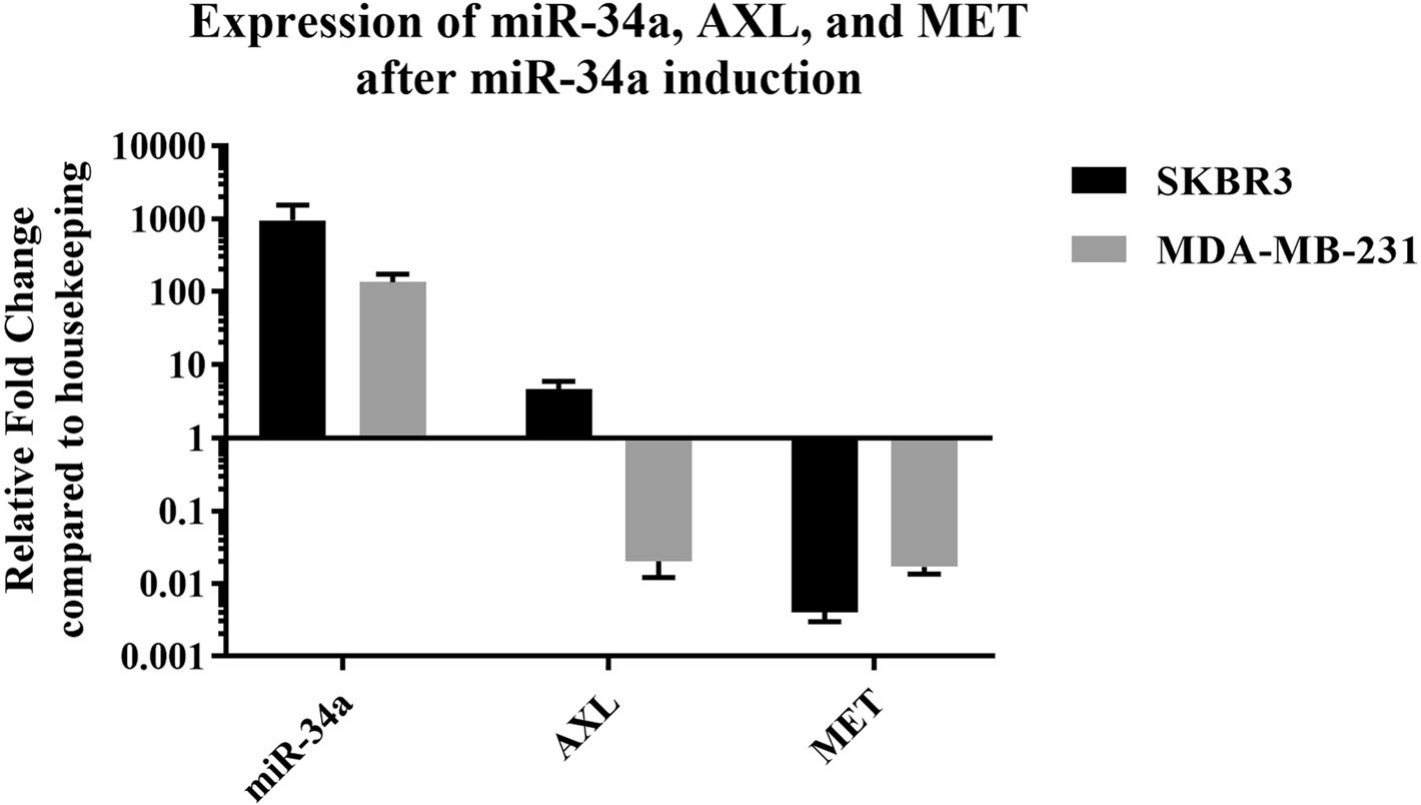
Expression of miR-34a, *AXL*, and *MET* in MDA-MB-231 and SK-BR-3 cell lines after miR-34a induction

After transduction and puromycin selection, GFP-positive cells were used for RNA extraction. The expression of miR-34a, *AXL*, and *MET* was evaluated in the cell lines after miR-34a induction using RT-qPCR (Fig. 4). All data were normalized to MCF-10A normal control cells.

### GEO dataset analysis

**Fig. 5:**
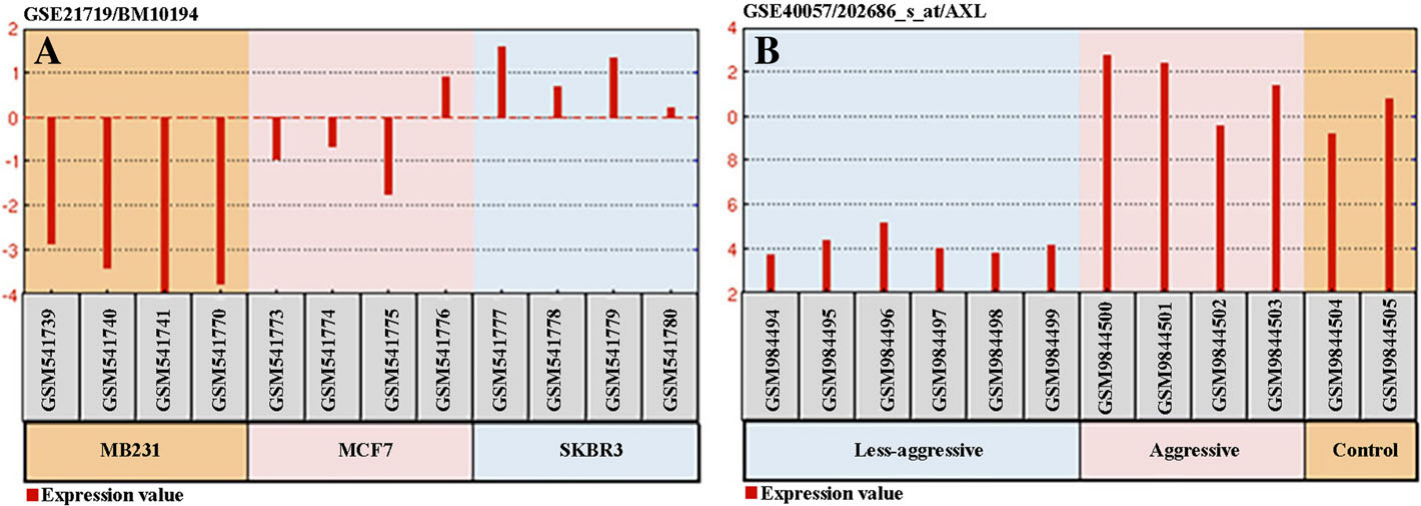
**a** MiR-34a expression before induction in SK-BR-3 and MDA-MB-231 cell lines (GSE21719). **b ***AXL* gene expression before miR-34a induction in SKBR3 and MDA-MB-231 cell lines (GSE40057)

Based on the data presented in GEO, GSE21719 microarray data indicated that miR-34a expression in SK-BR-3 and MDA-MB-231 cells was lower than that of normal control cells. Furthermore, the analysis of GSE40057 microarray data showed that *AXL* expression in MDA-MB-231 and SK-BR-3 cell lines was respectively higher and lower than that of normal control cell lines (Fig. 5a and b), which was in line with our results.

## Discussion

In the present study, bioinformatic databases including miRWalk, PicTar, and TargetScan were used. miR-34a was selected as the miRNA simultaneously targeting both *AXL* and *MET* 3′-UTRs. miR-34a targets oncogenes such as BCL-2, FRA-1, SIRT1, HER-2, and *MET*. It acts as a tumor suppressor miRNA [22] and plays a role in apoptosis and cell proliferation pathways [23, 24].

Previous studies highlight the role of *AXL* and *MET* in breast cancer. One of the principal factors in progression of cancers such as breast cancer is receptor tyrosine kinases, one of which is AXL, playing a part in PI3K and RAS signaling pathways. It has a great impact on development, invasion, epithelial-mesenchymal transition (EMT), and metastasis. The only known ligand of AXL is GAS6, inducing AXL phosphorylation and activation. This leads to its oncogenic function [25]. MET is also a receptor tyrosine kinase and encodes a cell surface protein that acts as a growth factor receptor and activates RAS, RAF, MEK, PTEN, AKT1, and mTOR. It has a great impact on cancer progression. Upon binding to its ligand, HGF, MET forms a homodimer, is phosphorylated, and is activated [26]. Activation of MET activates downstream oncogenes and results in cell proliferation. In addition, FAK and JNK are also activated, leading to gene rearrangement and invasion [27, 28]. Ho-Yen and colleagues showed that MET signaling has a direct role in the development and progression of breast cancer [12]. Cidadol et al. demonstrated that activation of the PI3K pathway induces AKT and mTOR expression and directly results in breast cancer cell proliferation and metastasis. They showed that PI3K mutations trigger the onset of the disease, and PI3K inhibitors decrease metastasis [29].

Bioinformatic analyses using miRWalk and miRBase databases in 2012 indicated that nearly 70% of miRNAs in vertebrates target at least one gene related to cell death or survival. Therefore, most miRNAs regulate apoptosis of cells [30, 31]. In 2005, the first report of 29 dysregulated miRNAs in breast cancer was published [14, 32]. Among these miRNAs were miR-17-92 and miR-34a, which were downregulated in various breast cancer subtypes [32, 33].

There is a direct relationship between the expression of miR-34a family and different cancers such as breast cancer [34]. In these cells, a marked decrease of pro-apoptotic miRNAs has been observed [35]. Furthermore, p53 induces the transcription of miR-215, miR-192, and miR-34 cluster [24, 36]. miR-34a, as a tumor suppressor and apoptosis-inducing miRNA, is directly activated by p53 [37, 38], which is dysregulated in most cancers. This locus is either deleted or methylated in cancers such as neuroblastoma, and hence it is not expressed [39]. Li et al. reported that the expression of miR-34a decreased in breast cancer. They observed that miR-34a was downregulated in five breast cancer cell lines including MDA-MB-231 [40]. Among the miR-34 cluster, miR-34a and miR-34c have similar seed regions [41].

In our study, after applying bioinformatic prediction software and tools, we also experimentally observed that miR-34a was downregulated in breast cancer cell lines, MDA-MB-231 and SK-BR-3, compared to normal breast cells (Fig. 2). Kaboli and colleagues found that miR-34a decreased in MDA-MB-231 and clinical samples [42]. In Liu and colleagues’ research, decreased expression of miR-34a/c was detected. Interestingly, systemic administration of miR-34a/c significantly inhibited tumor growth in animal models [43]. Paccezl et al. observed that *AXL* overexpression was related to cancer development and drug resistance, and they considered it as a target in acute myeloid lymphoma, breast cancer, and prostate cancer. Inhibition of AXL and its downstream proteins is a novel approach in treating cancers [44]. According to Cerachi et al., the expression of *AXL* in SK-BR-3 decreased while its expression in MDA-MB-231, HCT116, and H1299 was higher than that of SK-BR-3 [45, 46]. It is exactly in line with our findings in this study. *AXL* is targeted by several miRNAs, and its dysregulation has a role in various cancers. Elevated expression of *AXL* along with decreased miR-34a expression was observed in several cancer cell lines. AXL inhibition results in decreased cell proliferation and metastasis of breast and lung cancer cells [8, 25, 46]. In a study conducted by Ho-Yen, it was found that MET signaling was directly related to breast cancer progression, and anti-MET treatment proved effective for basal-like type 5 and TNBC type 6 breast cancers [12]. In 1997, Beviglia et al. observed increased expression of MET in MDA-MB-231 cells using the western blot method [47].

Our preliminary results indicated increased expression of *MET* and *AXL* in MDA-MB-231 cells compared with normal MCF-10A cells (Fig. 2). This strengthens the hypothesis that induction of miR-34a may reduce these oncogenes’ expression. In Beviglia’s study, after the induction of miR-34a, apoptosis was observed in MDA-MB-231 and SK-BR-3. In addition, in vitro overexpression of miR-34a led to the suppression of breast cancer stem cells. It also resulted in Notch1 mRNA degradation in MCF-7 cells [48]. However, they had not considered *MET* and *AXL* as the targets of miR-34a. Furthermore, induction of miR-34a induces the apoptosis of malignant peripheral nerve sheath tumor [36]. Mudduluru et al. found a negative correlation between AXL protein and miR-34a in a panel of lung, colon, and breast cancer samples. They also reported that by overexpressing miR-34a and miR-199a, the level of *AXL* mRNA and protein decreased in MDA-MB-231, HCT116, and H1299 cells [46]. These results indicate that the expression of AXL receptor is regulated by miR-34a and miR-199a/b, and their expression is controlled by promoter methylation in solid tumors. Our results were in line with their finding about the role of miR-34a in the regulation of *AXL* expression. However, we applied a bioinformatic approach, which is an inexpensive technique for the prediction of miRNAs. In a study in 2011, the researchers found that AXL mRNA was the target of several miRNAs, and its interaction with ectopically expressed miR-34a led to decreased luciferase activity in MCF-7 and MDA-MB-231 cells. In this study, AXL mRNA degradation led to decreased AXL protein expression and decreased phosphorylation of AKT. Targeting AXL with miR-34a inhibited cell migration but had no effect on cell proliferation [8]. Another study showed that miR-34a expression level was low in MDA-MB-231 cells, but cell death was induced after miR-34a overexpression [49]. However, we did not note any significant difference in cell viability after miR-34a induction in breast cancer cell lines.

**Fig. 6:**
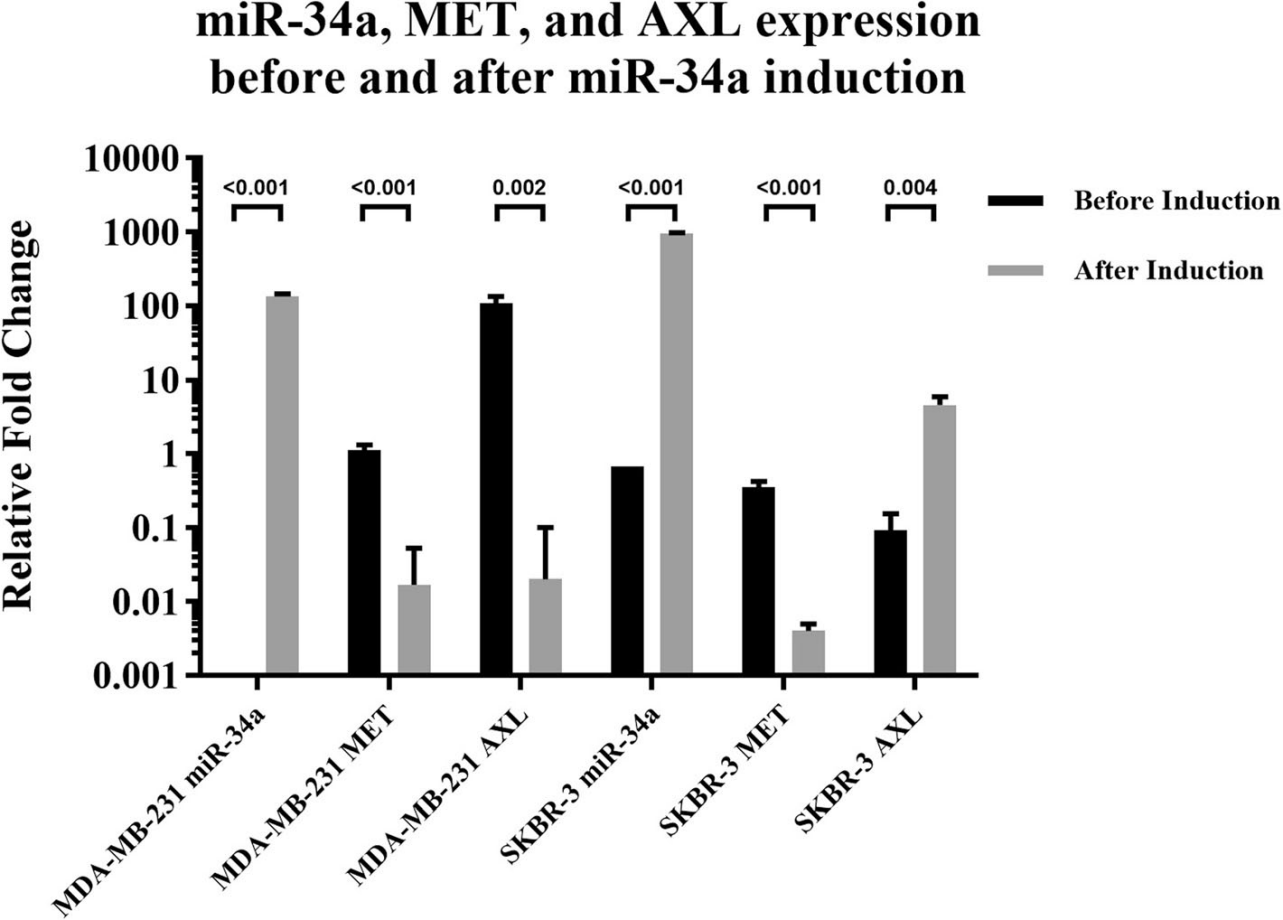
Comparison of miR-34a, *MET*, and *AXL* expression before and after miR-34a induction in the cell lines. Results showed that, after miR-34a induction, *MET* and *AXL* expression decreased in MDA-MB-231 cells. In SK-BR-3 cells, *MET* expression decreased, but *AXL* expression increased

Based on previous studies, we hypothesized that we can decrease the expression of *AXL* and *MET* simultaneously by overexpressing miR-34a in breast cancer cell lines. As indicated, we evaluated basal expression of these genes, and it was lower than that of the normal breast cell line as expected. By overexpressing miR-34a using a viral vector, the expression of *MET* and *AXL* was altered. Both *AXL* and *MET* expression decreased in MDA-MB-231 cells, but in SK-BR-3 cells, *AXL* expression increased while *MET* expression declined (Fig. 6).

We also analyzed GSE21832 and found that *AXL* expression in MDA-MB-231 and SK-BR-3 cells was respectively higher and lower than that of normal cells, which was in line with our results. Analyzing GSE21719 indicated that the expression of miR-34a in MDA-MB-231 cells was lower than that of normal control cells, which is in agreement with our results.

Based on Leisheng’s studies using RT-qPCR, miR-34a expression was lower than that of normal controls in 13 breast cancer cell lines including MDA-MB-231 and SK-BR-3 [40]. In our research, we observed that, compared to the normal cells, *MET* expression was higher (1.12 folds) in MDA-MB-231 cells although it was statistically insignificant (*p* value> 0.05). *MET* expression in the SK-BR-3 cell line was lower than that of normal cells (*p* value < 0.05). After induction of miR-34a expression, similar results were obtained compared to other studies in that *MET* and *AXL* expression decreased in MDA-MB-231 cells (Fig. 6). Overall, our results were in line with the mentioned study’s results: induction of miR-34a expression results in decreased expression of *AXL* and *MET* in MDA-MB-231 cells.

Our results indicated that the expression of *AXL* was higher than that of *MET* before miR-34a induction in MA-MB-231 cells. After miR-34a induction, *AXL* expression decreased more than that of *MET*. This is probably due to fact that the free energy of miR-34a:*AXL* 3′-UTR interaction is higher than that of miR-34a:*MET* 3′-UTR interaction. Furthermore, the seed match between *AXL* 3′-UTR and miR-34a is 8mer, and it is 7mer-8 m for *MET* 3′-UTR and miR-34 interaction.

Regarding *AXL* expression in SK-BR-3 cells after miR-34a induction, we did not find any reports. Mackiewicz et al. did not overexpress miR-34a in SK-BR-3 cells since at the beginning of the study the researchers found that *AXL* expression was low, and they intended to study cell lines in which both *MET* and *AXL* expression was high. In our study, RT-qPCR results showed that *MET* expression decreased after miR-34a induction in SK-BR-3 cells. On the other hand, *AXL* expression increased after induction. This was probably due to direct or indirect interaction of miR-34a with AXL inhibitors. SK-BR-3 is a HER2^+^ cell line in which ADAM metalloproteinases are highly expressed. ADAM10 expression results in HER2 ectodomain sheddase activity [50]. In addition, ADAM10 and ADAM17 are AXL inhibitors. They downregulate AXL downstream signaling by proteolytic cleavage of the AXL extracellular domain [51]. Since we observed *AXL* overexpression after miR-34a induction, we explored miR-34a and ADAM10 interaction in TargetScan. There are two conserved targets for miR-34a in the 3′-UTR of ADAM10. Therefore, it can be inferred that induction of miR-34a leads to ADAM10 repression and increased expression of *AXL* in SK-BR-3 cells.

There are various studies on *AXL* in several cancers such as lung cancer. For example, luciferase assay results indicate that miR-34a targets 3′-UTR of *AXL* [44]. Most studies have been conducted to inhibit highly expressed oncogenes in cancer cell lines such as HER2 in breast cancer [52]. Most studies aim to target HER2 in the SK-BR-3 cell line since SK-BR-3 highly expresses HER2 [53]. Since *AXL* expression is low or absent in SK-BR-3 cells, there is not any research targeting *AXL* with miR-34a. However, Leisheng et al. overexpressed miR-34a in several TNBC (MDA-MB-231 and MDA-MB-468) and HER2^+^ (BT474 and SK-BR-3) cell lines and assessed its effect in vitro and in vivo. They reported that miR-34a overexpression significantly decreased cell proliferation, migration, and invasion. In addition, it increased apoptosis in MDA-MB-231 and SK-BR-3 cells [40]. Comparing these results with ours, it can be inferred that miR-34a overexpression in SK-BR-3 cells may decrease *AXL* expression directly or indirectly.

## Conclusion

In the present study, using a bioinformatic approach, miR-34a was selected as the miRNA targeting both *MET* and *AXL* simultaneously. miR-34a expression was decreased in MDA-MB-231 and SK-BR-3 cells compared with normal control cells. Upon miR-34a induction, both *MET* and *AXL* expression decreased in MDA-MB-231. *MET* expression decreased while *AXL* expression increased in SK-BR-3 cells. This phenomenon indicates that expression of these oncogenes is closely related to cell type and content. Therefore, miR-34a can be used for the treatment of at least some subtypes of breast cancer. In addition, since miR-34a expression is low in HER2^+^ and TNBC cells, it can be used as a diagnostic or therapeutic agent in these breast cancer types. However, more cell lines, clinical samples, and animal models should be evaluated to understand whether miR-34a can be used as a therapeutic agent for breast cancer.

## Abbreviations

3′-UTR: Three prime untranslated region
cDNA: Complementary DNA
DMEM: Dulbecco’s modified Eagle medium
EMT: Epithelial-mesenchymal transition
FBS: Fetal bovine serum
GEO: Gene Expression Omnibus
HS: Horse serum
miRNAs: microRNAs
PI3K: Phosphatidylinositol-3-kinase
RTKs: Receptor tyrosine kinases
RT-qPCR: Quantitative real-time PCR
SNORD47: Small nuclear RNA 47
TNBC: Triple-negative breast cancer
